# Real-time observation of microcirculatory leukocytes in patients undergoing major liver resection

**DOI:** 10.1038/s41598-021-83677-0

**Published:** 2021-02-25

**Authors:** Zühre Uz, C. Ince, L. Shen, B. Ergin, T. M. van Gulik

**Affiliations:** 1 Department of Surgery, Cancer Center Amsterdam, Amsterdam UMC, Academic Medical Center, University of Amsterdam, Meibergdreef 9, 1105 AZ Amsterdam, The Netherlands; 2 Department of Translational Physiology, Amsterdam UMC, Location Academic Medical Center, University of Amsterdam, Meibergdreef 9, 1105 AZ Amsterdam, The Netherlands; 3 Department of Intensive Care Adults, Erasmus MC, Doctor Molewaterplein 40, 3015 GD Rotterdam, The Netherlands

**Keywords:** Physiology, Gastroenterology

## Abstract

Ischemia/reperfusion injury and inflammation are associated with microcirculatory dysfunction, endothelial injury and glycocalyx degradation. This study aimed to assess microcirculation in the sublingual, intestinal and the (remnant) liver in patients undergoing major liver resection, to define microcirculatory leukocyte activation and its association with glycocalyx degradation. In this prospective observational study, the microcirculation was assessed at the beginning of surgery (T0), end of surgery (T1) and 24 h after surgery (T2) using Incident Dark Field imaging. Changes in vessel density, blood flow and leukocyte behaviour were monitored, as well as clinical parameters. Syndecan-1 levels as a parameter of glycocalyx degradation were analysed. 19 patients were included. Sublingual microcirculation showed a significant increase in the number of rolling leukocytes between T0 and T1 (1.5 [0.7–1.8] vs*.* 3.7 [1.7–5.4] Ls/C-PCV/4 s respectively, *p* = 0.001), and remained high at T2 when compared to T0 (3.8 [3–8.5] Ls/C-PCV/4 s, *p* = 0.006). The microvascular flow decreased at T2 (2.4 ± 0.3 vs*.* baseline 2.8 ± 0.2, respectively, *p* < 0.01). Duration of vascular inflow occlusion was associated with significantly higher numbers of sublingual microcirculatory rolling leukocytes. Syndecan-1 increased from T0 to T1 (42 [25–56] vs*.* 107 [86–164] ng/mL, *p* < 0.001). The microcirculatory perfusion was characterized by low convection capacity and high number of rolling leukocytes. The ability to sublingually monitor the rolling behaviour of the microcirculatory leukocytes allows for early identification of patients at risk of increased inflammatory response following major liver resection.

## Introduction

Liver resection (LR) remains the only curative option for liver malignancies^1^. Systemic inflammatory response as a result of ischemia reperfusion injury (IRI) during vascular inflow occlusion (VIO), surgical trauma and preoperative co-morbidities may contribute to poor postoperative outcomes following LR^2–7^. Liver IRI induces local expression of proinflammatory cytokines, promoting the recruitment and activation of Kupffer cells (KC). Activated KC are signalled for transmigration into the liver parenchyma where they release reactive oxygen species contributing to oxidative stress in the liver as well as in distant organs^8^. During the late phase of inflammation, cytokine production spills into the systemic circulation resulting in global activation of neutrophils and lymphocytes. Studies have shown that apart from the liver, multiple organs are affected by this process such as; myocardium, pancreas, intestines, kidneys, adrenal glands and the lungs^8^.

Leukocyte activation in multiple organs occurs at the endothelial barrier of the microvasculature where oxidative stress, decreased nitrous oxide production and a surge in pro-inflammatory cytokine production result in accumulation of activated leukocytes and degradation of endothelium^9^. Studies have shown that degradation of the endothelium results in capillary leakage and disruption of capillaries^10,11^. The glycocalyx is the anti-adhesive layer of the endothelium, whilst the disruption of the glycocalyx stimulates firm adhesion of the leukocytes^12^.

The assessment of leukocyte–endothelial interactions within the microcirculation becomes a potential target for therapeutic interventions^13,14^. Microcirculatory bedside monitoring has been made possible through the introduction of handheld vital microscopes (HVM). These devices are to date, predominately applied sublingually for microvascular monitoring in several disease states^15^. There is now abundant literature on the use of HVM in abdominal microcirculatory monitoring, demonstrating its feasibility for intraoperative use^16–20^. Our research group validated a microcirculatory leukocyte quantification methodology, using the third generation of HVM, the Incident Dark field Imaging (IDFI)^21^. Using this methodology, it is possible to monitor leukocyte recruitment and activation within the microcirculation in a non-invasive manner.

We hypothesized that an increase in microcirculatory and endothelial dysfunction due to IRI during liver resection, is related to glycocalyx degradation and the number of activated leukocytes. It is anticipated that the rolling leukocytes indicate the combined effect of leukocyte activation and endothelial injury through glycocalyx degradation. The sublingual area presents a novel target for the perioperative monitoring of leukocyte activation, owing to its non-invasive accessibility in patients^22–25^.

The aim of this study was to assess microcirculatory alterations, behaviour of leukocytes and their association with glycocalyx degradation in patients undergoing major hepatectomy exposed to IRI. We therefore assessed microcirculatory alterations in terms of capillary density and flow in the sublingual space as compared with liver and intestine as distant, intra-abdominal organ sites.

## Results

### Patient demographics and hemodynamic parameters

A total of 19 patients were recruited for the study; the main demographics and surgical characteristic are shown in Table 1. In 16 of the 19 patients, VIO was applied; the cumulative VIO duration was calculated for the whole group (33.5 ± 20 min), see Table 1. Morbidity occurred in 14 patients within 30 days after surgery, of which 12 had a major complication (Clavien–Dindo ≥ 3). Two patients died within 30 days postoperatively in the ICU department, due to sepsis and multiple organ failure.

**Table 1:** Subject demographics, pathology, surgical profile and postsurgical phase.

Patient demographics	Mean ± SD
Age (years)	61.3 ± 12.1
Weight (kg)	73.5 ± 15
BMI (*kg*/m^2^)	24.9 ± 4.3
Gender (male:female)	11: 8
**Co-morbidities**	
Smoker (Y:N)	3: 16
COPD (Y:N)	3: 16
Hypertension (Y N)	9: 10
Diabetes (Y:N)	3: 16
**Pathology**	
Perihilar carcinoma (*n*)	8
HCC (*n*)	2
CRLM (*n*)	2
Gallbladder carcinoma (*n*)	1
Haemangioma (*n*)	1
Intrahepatic cholangiocarcinoma (*n*)	4
Biliary Ig4-related disease (*n*)	1
**Type of surgery**	
Left hemi-hepatectomy (*n*)	4
Right hemi-hepatectomy (*n*)	14
ALPPS 1st phase (*n*)	1
**Surgical characteristics**	
Surgical duration (mins)	486 ± 121
Blood loss (mLs)	1674 ± 875
VIO (n)	1.4 ± 0.8
VIO duration (mins)	33.5 ± 20
VIO applied (Y:N)	16: 3
**Postsurgical phase**	
24-h morbidity (Y:N)	6: 13
30-day morbidity (Y:N)	14: 5
Clavien–Dindo ≥ 3 within 30 days (Y:N)	12: 7
**Clavien–Dindo scoring within 30 days**	
Score 0 (number (%))	3 (16)
Score 1 (number (%))	3 (16)
Score 2 (number (%))	1 (5)
Score 3 (number (%))	5 (26)
Score 4 (number (%))	5 (26)
Score 5 (number (%))	2 (11)
30-day mortality (Y:N)	2*: 17

*BMI* body mass index, *ALPPS* Associating Liver Partition and Portal Vein Ligation for Staged hepatectomy, *COPD* chronic obstructive pulmonary disease, *HCC* hepatocellular carcinoma, *CRLM* colorectal liver metastasis, *VIO* vascular inflow occlusion. *Mortality due to sepsis.

The systemic hemodynamic parameters were stable throughout the intraoperative period and improved 24 h after surgery. There were no significant changes in patient temperature or in SpO_2_ throughout the study period, see Table 2. A significant increase in lactate concentration was observed from T0 to T1 (1.6 ± 0.9 vs*.* 2.8 ± 1.1 mmol/L respectively, *p* < 0.005) during surgery. Lactate levels remained increased at the same level at T2 (Table 2).

**Table 2:** Patient systemic hemodynamic and arterial blood gas results.

	T0	T1	T2
**Systemic hemodynamic**			
Heart rate (bpm)	72 ± 15	72 ± 16	83 ± 18*
Blood pressure systolic (mmHg)	107 ± 17	100 ± 16	124 ± 25*^,+^
Blood pressure diastolic (mmHg)	57 ± 13	50 ± 6*	73 ± 16*^,+^
Mean arterial pressure (mmHg)	74 ± 12	67 ± 6*	87 ± 18^+^
Stroke volume (mLs)	71.6 ± 26.5	65.7 ± 17.6	X
SpO_2_ (%)	99.7 ± 0.8	99.9 ± 0.5	96 ± 2.6*^,+^
Temperature (°C)	35.8 ± 1.7	36.7 ± 0.5	36.7 ± 0.5
**Arterial blood gas**			
Haemoglobin (mmol/L)	7.9 ± 0.9	6.9 ± 1.1*	6.9 ± 1.2*
pH	7.4 ± 0.1	7.4 ± 0.1	7.4 ± 0.1
PCo_2_ (kPa)	6.1 ± 2.6	5.8 ± 0.6	5.4 ± 0.6
PO_2_ (kPa)	29.4 ± 21.7	29 ± 20.2	11.5 ± 4.9*^,+^
HbO_2_ (%)	97.5 ± 1.66	97 ± 2.7	88.6 ± 12.7*^,+^
Glucose (mmol/L)	8.2 ± 2.3	10 ± 2.3*	7.6 ± 2.7^+^
Lactate (mmol/L)	1.6 ± 0.9	2.8 ± 1.1*	2.8 ± 2*

*T0* at the beginning of surgery, *T1* at the end of surgery, *T2* 24 h after surgery. Values are represented as mean ± SD, *p < 0.05 compared to baseline T0, ^+^*p* < 0.05 compared to T1.

The liver enzymes, ALT and AST were significantly increased (ALT: 105 ± 208 at T0 vs. 631 ± 420 IU/L at T1, *p* < 0.005. AST: 124 ± 210 at T0 vs. 549 ± 309 IU/L at T1, *p* < 0.005) at 24 h after surgery (Table 3).

**Table 3:** Liver function blood results.

	T0	T1	T2
**Liver function enzymes**			
AST (IU/L)	105 ± 208	X	631 ± 420*
ALT (IU/L)	124 ± 210	X	549 ± 309*
ALP (IU/L)	308 ± 254	X	162 ± 105*
GGT (IU/L)	434 ± 456	X	237 ± 176
Bilirubin (µmol/L)	21 ± 22	X	29 ± 18

*T0* at the beginning of surgery, *T1* at the end of surgery, *T2* 24 h after surgery, *AST* aspartate aminotransferase, *ALT* alanine aminotransferase, *ALP* alkaline phosphatase test, *GGT* gamma-glutamyl transferase. Values are represented as mean ± SD, *p < 0.05 compared to T0.

## Microcirculatory parameters

### Microcirculation in different organ surfaces

**Figure 1:**
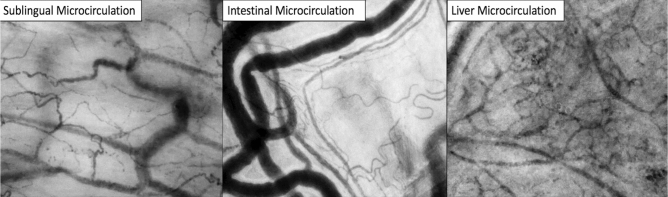
Screenshots of sublingual, intestinal and liver microcirculation, respectively, obtained by incident dark field handheld video microscopy. Dimensions of the field of view of the microcirculatory images is 1.55 mm × 1.16 mm.

Images of sublingual, intestinal and liver organ microcirculation are shown in Fig. 1. This figure shows screenshots of the video-clips of sublingual, intestinal and hepatic microcirculation, obtained with Cytocam handheld video microscopy, in the same patient at baseline. The sublingual microcirculation shows capillaries, post-capillary venules and large veins. The vessels with the smallest diameter are the capillaries, the post-capillary venules are the vessels distal to the capillaries with a larger diameter when compared to the capillaries. The post-capillary venules are merging into larger venules in the left/down corner. The intestinal area shows a brighter background when compared to the sublingual and hepatic areas, with less capillaries. The large venules are seen on the left side of the image, the capillaries are more seen on the right side of the image. The intestinal capillaries are curly, whereas the post-capillary venules in which the capillaries merge into, are straighter. The hepatic microcirculation shows a darker background when compared to sublingual or intestinal microcirculation. Due to the rich sinusoidal microvasculature, the hepatic microcirculation demonstrates a high vessel density in the image. The sinusoidal microvessels are making shorter distances with more interconnections to the other sinusoidal microvessels as compared to the sublingual and intestinal capillaries.

### Sublingual microcirculatory flow and density parameters

**Figure 2:**
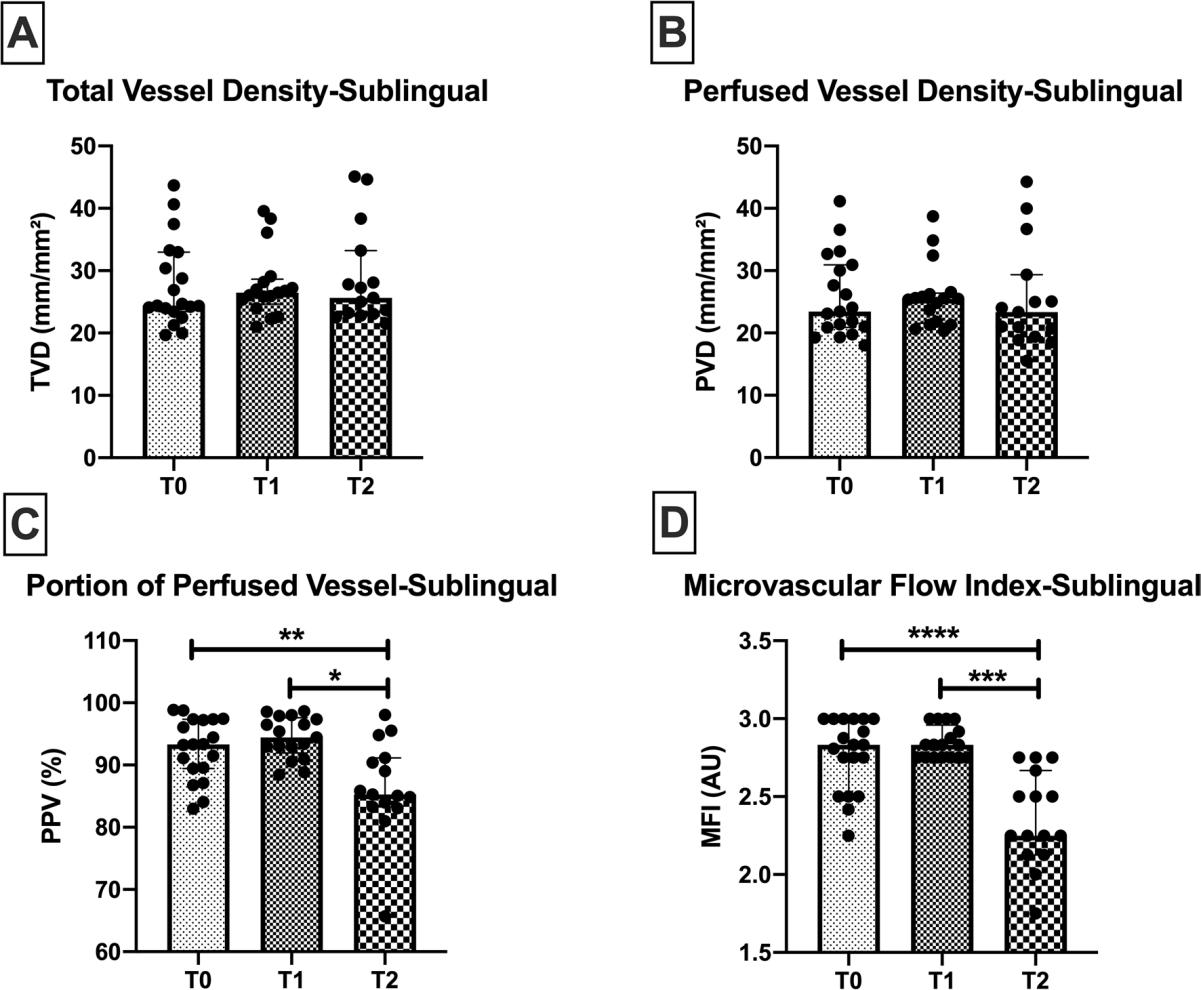
Sublingual microcirculation. Sublingual microcirculatory parameters at the beginning of surgery (T0), end of surgery (T1) and 24 h after surgery (T2) in 19 patients. Sublingual portion of perfused vessel and microvascular flow index (MFI) decrease 24 h after surgery. *TVD* total vessel density, *PVD* perfused vessel density, *PPV* portion of perfused vessel, *MFI* microvascular flow index. Values are represented as median [interquatile] for TVD, PVD and MFI and mean ± SD for PPV, *p < 0.05, **p < 0.01, ***p < 0.001, ****p < 0.0001.

At T1, the end of surgery, there were no significant changes in sublingual microcirculatory density (Fig. 2A,B) or flow (Fig. 2C,D) parameters. No differences were found in TVD or PVD during 24 h (Fig. 2A,B). A decrease in PPV was observed at T2 when compared to T0 and T1 (T2: 86.4 ± 7.6% vs*.* T0: 92.6 ± 4.9%, *p* = 0.008 and T2: 86.4 ± 7.6% vs*.* T1: 94.3 ± 3.3%, *p* = 0.01) (Fig. 2C)*.* Similarly, a decrease in MFI was also observed at T2 when compared to T0 and T1 (T2: 2.2 [2.1–2.6] vs*.* T0: 2.8 [2.5–3], *p* = 0.0001. and T2: 2.2 [2.1–2.6] vs*.* T1: 2.8 [2.7–2.9], *p* = 0.0004 (Fig. 2D). These results indicate a reduction of microvascular blood flow occurring 24 h after surgery, while no significant differences were observed concerning the microvascular density parameters expressed by TVD and the PVD.

### Intestinal microcirculatory flow and density parameters

**Figure 3:**
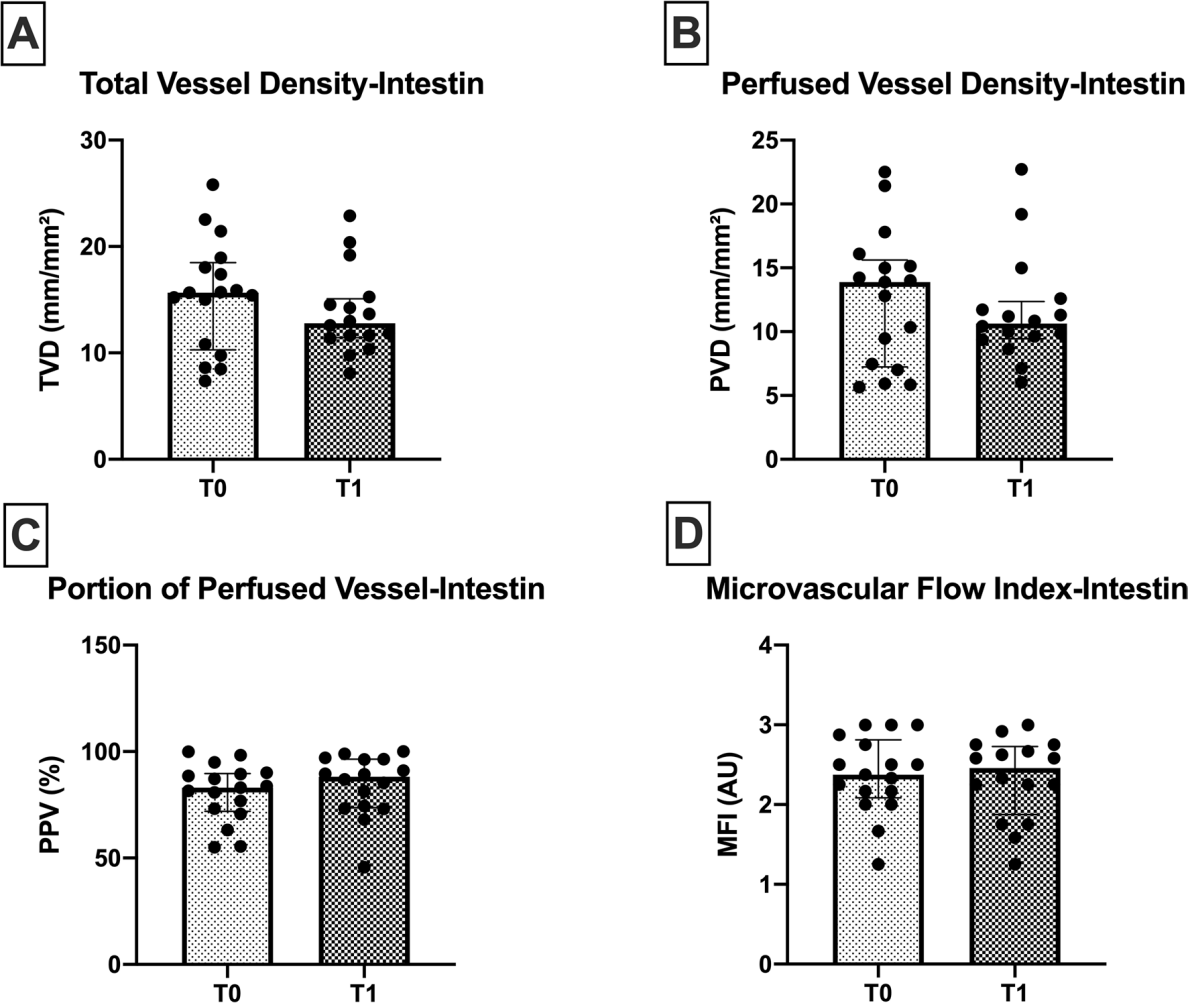
Intestinal microcirculation. Intestinal microcirculatory parameters at the beginning of surgery (T0) and end of surgery (T1) in 18 patients. No significant differences were observed in microcirculatory density nor flow in the perioperative period. TVD; total vessel density. PVD; perfused vessel density. PPV; portion of perfused vessel. MFI; microvascular flow index. Values are represented as median [interquatile] for PVD and PPV and mean ± SD for TVD and MFI.

No significant differences were observed in any of the microcirculatory parameters at the intestinal level (Fig. 3). The TVD, PVD, and MFI showed a trend in decreasing values at the end of surgery (T1).

### Hepatic microcirculatory flow and density parameters

**Figure 4:**
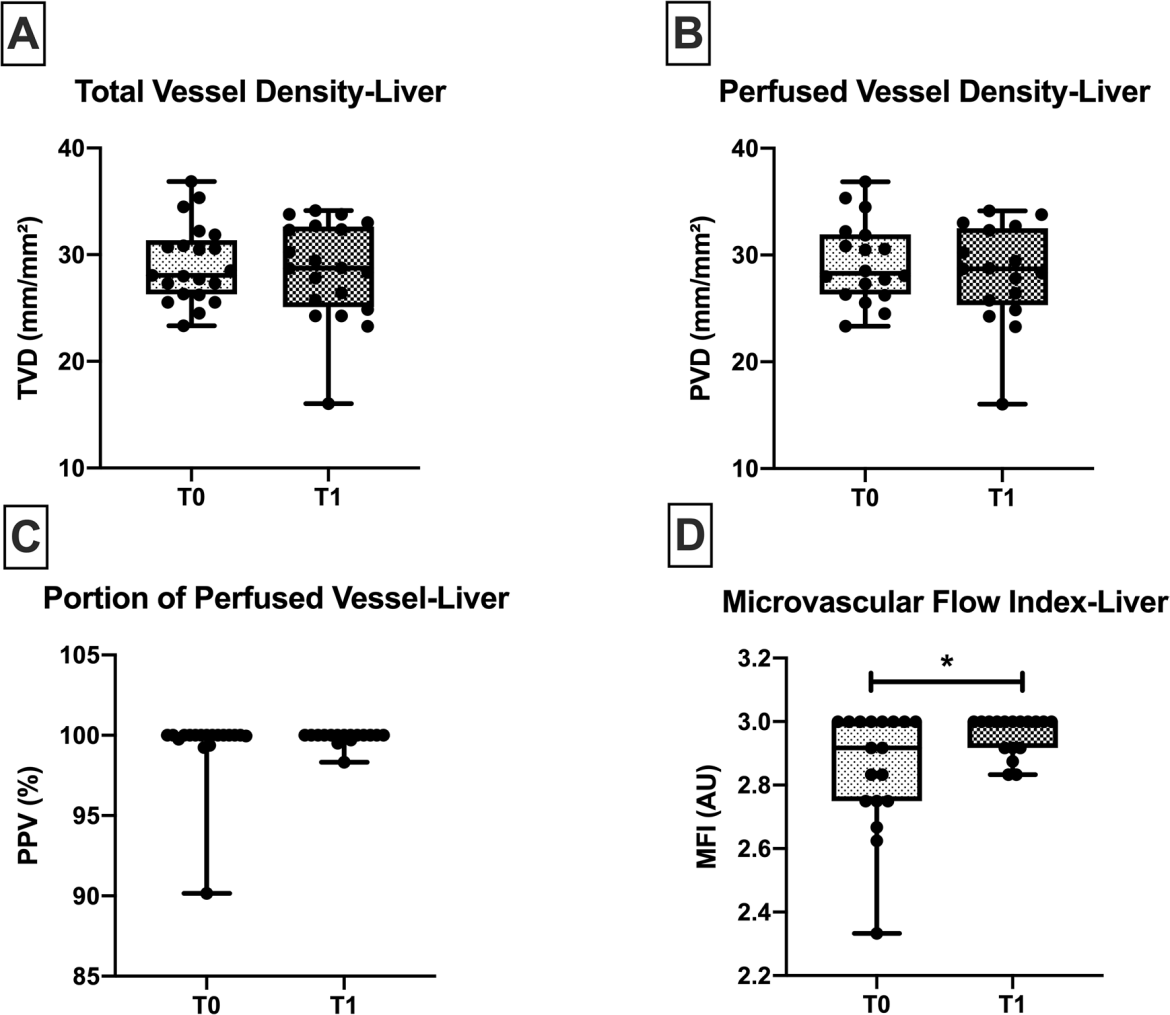
Liver microcirculation. Liver microcirculatory parameters at the beginning of surgery (T0) and end of surgery (T1). A significant increase was found in the hepatic MFI from T0 to T1 in 18 patients. TVD; total vessel density. *PVD* perfused vessel density, *PPV* portion of perfused vessel, *MFI* microvascular flow index. Values are represented as median [interquatile] for PVD and MFI and mean ± SD for TVD and PVD, *p < 0.05.

No significant differences were observed in microcirculatory density parameters and PPV on the liver level at any study time-points (Fig. 4). Only a significant increase was found in the hepatic MFI, from T0 to T1 (2.9 [2.75–3.00] vs*.* 3.0 [2.92–3.00], *p* = 0.02) (Fig. 4D). The measured liver segment was part of the future remnant liver segment, and after resection, received all the portal vein volume which normally is distributed throughout the whole liver parenchyma.

### Sublingual microcirculatory leukocytes count

**Figure 5:**
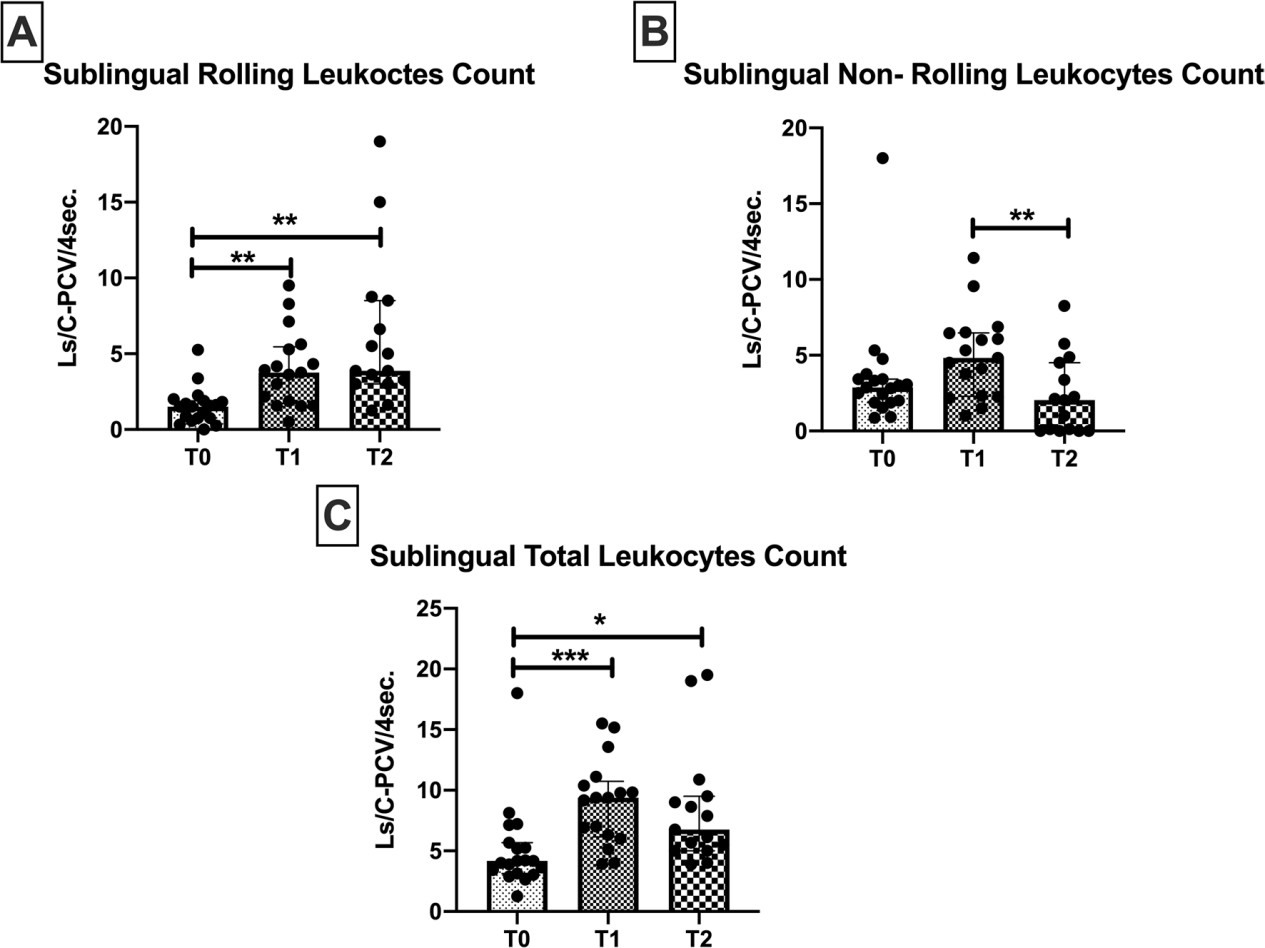
Leukocyte quantification in the sublingual area assessed at the beginning of surgery (T0), end of surgery (T1) and 24 h after surgery (T2). The microcirculatory rolling leukocytes count increased during the time course. Values are represented as median [interquatile] *p < 0.05, **p < 0.01, ***p < 0.001.

The number of rolling leukocytes increased from T0 to T1 (1.5 [0.7–1.8] vs*.*3.7 [1.7–5.4] Ls/C-PCV/4 s respectively, *p* = 0.001), and remained high at T2 when compared to T0 (3.8 [3–8.5] vs*.* 1.5 [0.7–1.8] Ls/C-PCV/4 s respectively, *p* = 0.006) but not when compared to T1 (Fig. 5A). The number of non-rolling leukocytes in the sublingual microcirculation subsequently decreased at T2 when compared to T1 (2 [0–4.5] vs*.* 4.8 [2.2–6.4] Ls/C-PCV/4 s, respectively, *p* = 0.002), to levels not significantly different from baseline (Fig. 5B). The inverse behaviour displayed by the non-rolling and rolling leukocytes is indicative of a shift in leukocyte behaviour as leukocytes become activated in the post-operative period. The total sublingual leukocyte counts significantly increased from T0 to T1 (4.1 [3.1–5.6] vs*.* 9.3 [6.1–10.7] Ls/C-PCV/4 s respectively, *p* = 0.0004), and remained elevated at T2 when compared to T0 (6.7 [5–9.5] vs*.* 4.1 [3.1–5.6] Ls/C-PCV/4 s respectively, *p* = 0.01) (Fig. 5C).

### Intestinal leukocytes count

**Figure 6:**
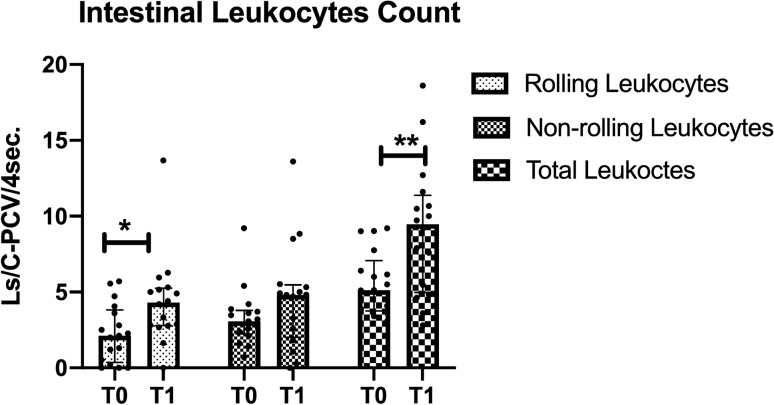
Leukocyte quantification in the intestinal serosa assessed at the beginning of surgery (T0) and end of surgery (T1). The microcirculatory rolling leukocytes count increased during surgery. Values are represented as median [interquatile], *p < 0.05, **p < 0.01.

An increase in the number of rolling leukocytes was observed in the intestinal serosa at T1 when compared to T0 (4.4 [2.7–5.2] vs*.* 2.1 [0.3–3.8] Ls/C-PCV/4 s respectively, *p* = 0.04). Also, an increase in the total leukocyte quantification was detected from T0 to T1 (5.1 [3.7–7] vs*.* 9.4 [4.9–11.3] Ls/C-PCV/4 s respectively, *p* = 0.001) (Fig. 6).

### Systemic leukocytes count

**Figure 7:**
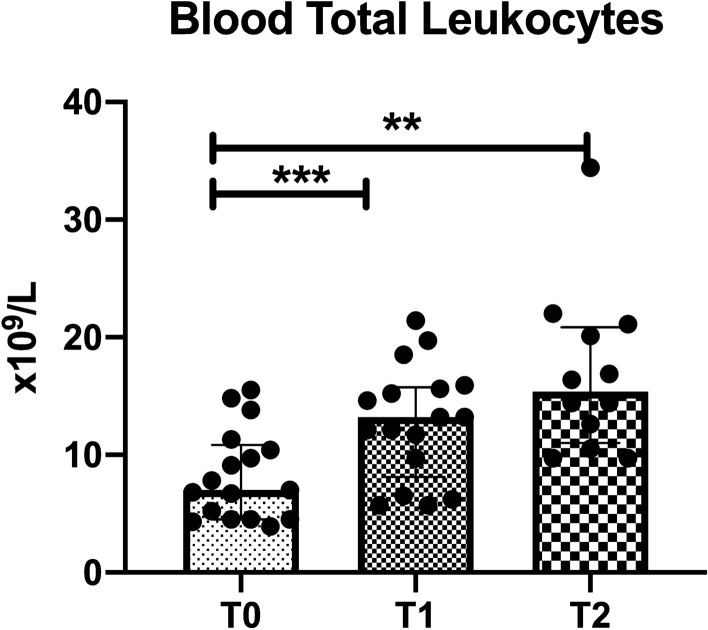
Leukocyte quantification within the systemic circulation (c) assessed at the beginning of surgery (T0), end of surgery (T1) and 24 h after surgery (T2). The systemic white blood cell count increased during the time course. Values are represented as mean ± SD, **p < 0.01, ***p < 0.001.

The increase observed in sublingual microcirculatory leukocytes was corroborated by an increase in systemic white blood cell counts (WBC) from T0 to T1 (8.2 ± 3.8 vs*.* 12.7 ± 4.8 × 10^9^ L, respectively, *p* = 0.0009). The increase in systemic WBC remained elevated at T2, at the same level when compared to T0 (16.8 ± 6.9 vs*.* 8.2 ± 3.8 × 10^9^ L, respectively, *p* = 0.003) (Fig. 7). These findings show that there was no significant increase of the systemic leukocytes between the end of surgery at T1 and 24 h later at T2.

### Effects of cumulative duration of VIO on microcirculation: no VIO, short duration VIO and long duration VIO

In 3 patients, no VIO was applied. In 5 patients, VIO duration was 30 min or shorter. In the other 11 patients, VIO duration was longer than 30 min, going up to 68 min of total occlusion time. In order to analyse the effects of long duration ischemia and no or short ischemia, we split the patients in two groups: (1) short-VIO (no VIO and short VIO duration (0–30 min, n = 8) and (2) long-VIO (VIO duration 31 min or longer, n = 11). The patients in the long-VIO group showed significantly higher numbers of sublingual rolling leukocytes when compared to the short-VIO group at the end of surgery at T1 (short-VIO 2.7 ± 1.2 vs*.* long-VIO 5 ± 2.8, *p* < 0.05). Lactate levels were significantly higher in the long-VIO group as compared to short-VIO 24 h after surgery at T2 (short-VIO 1.6 ± 0.8 vs*.* long-VIO 3.6 ± 2.2, *p* < 0.05). Other parameters such as MFI, Syndecan-1 levels and duration of VIO did not show any significant differences between patients in the short-VIO or long-VIO groups (Table 4).

**Table 4:** Higher rolling leukocytes and lactate values in patients with long vascular inflow occlusion (VIO) duration.

	Group 1: cumulative short duration VIO (0–30 min) N = 8	Group 2: cumulative long duration VIO (31–70 min) N = 11	p value
**Lactate (mmol/L)**			
T0	1.4 ± 0.5	1.8 ± 1.2	0.464
T1	2.4 ± 1	3.1 ± 1.1	0.175
T2	1.6 ± 0.8	3.6 ± 2.2	0.047
**MFI (AU)**			
T0	2.8 ± 0.2	2.8 ± 0.3	0.874
T1	2.8 ± 0.1	2.9 ± 0.1	0.523
T2	2.5 ± 0.3	2.3 ± 0.3	0.200
**Rolling leukocytes (Lc/c-pcv/4 s)**			
T0	1.3 ± 1.1	1.9 ± 1.3	0.402
T1	2.7 ± 1.2	5 ± 2.8	0.049
T2	3.3 ± 1.8	9.1 ± 6.1	0.064
**Syndecan-1 levels (ng/mL)**			
T0	37 [12–57]	48 [30–80]	0.274
T1	118 [93–181]	91 [71–130]	0.190
T2	151 [90–277]	162 [94–240]	0.958
**Surgery duration (min)**			
T0–T1	478 ± 127	449 ± 130	0.670

*T0* at the beginning of surgery, *T1* at the end of surgery, *T2* 24 h after surgery, *MFI* microvascular flow index.

### Relationship of glycocalyx degradation and activated sublingual microcirculatory leukocytes

**Figure 8:**
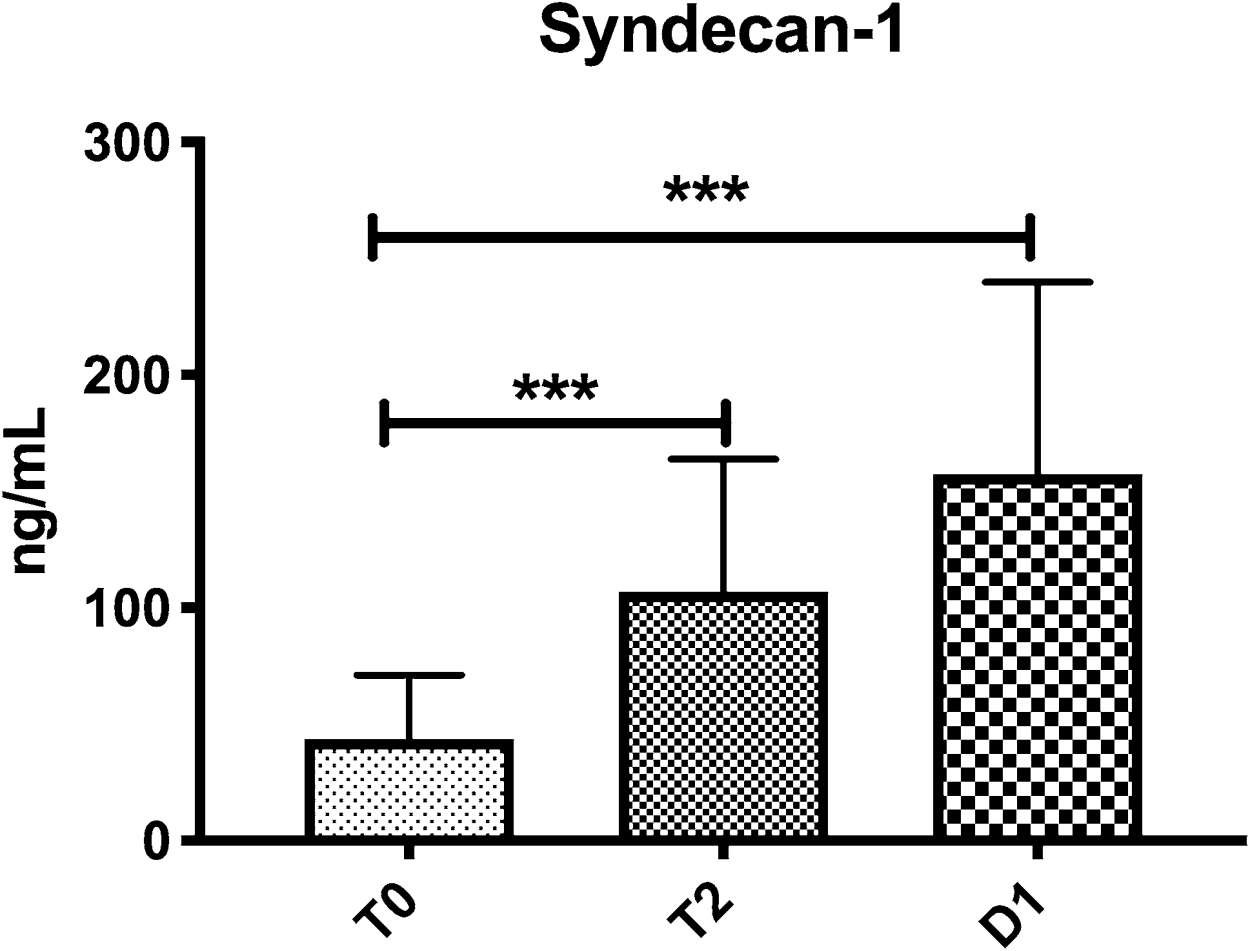
Syndecan-1 levels measured in blood at the beginning of surgery (T0), the end of surgery (T1) and 24 h later (T2). Syndecan-1 was increased at the end of surgery (T1) and this increase persisted at T2 (24 h). Values are represented as median [interquartile, ***p < 0.001.

**Figure 9:**
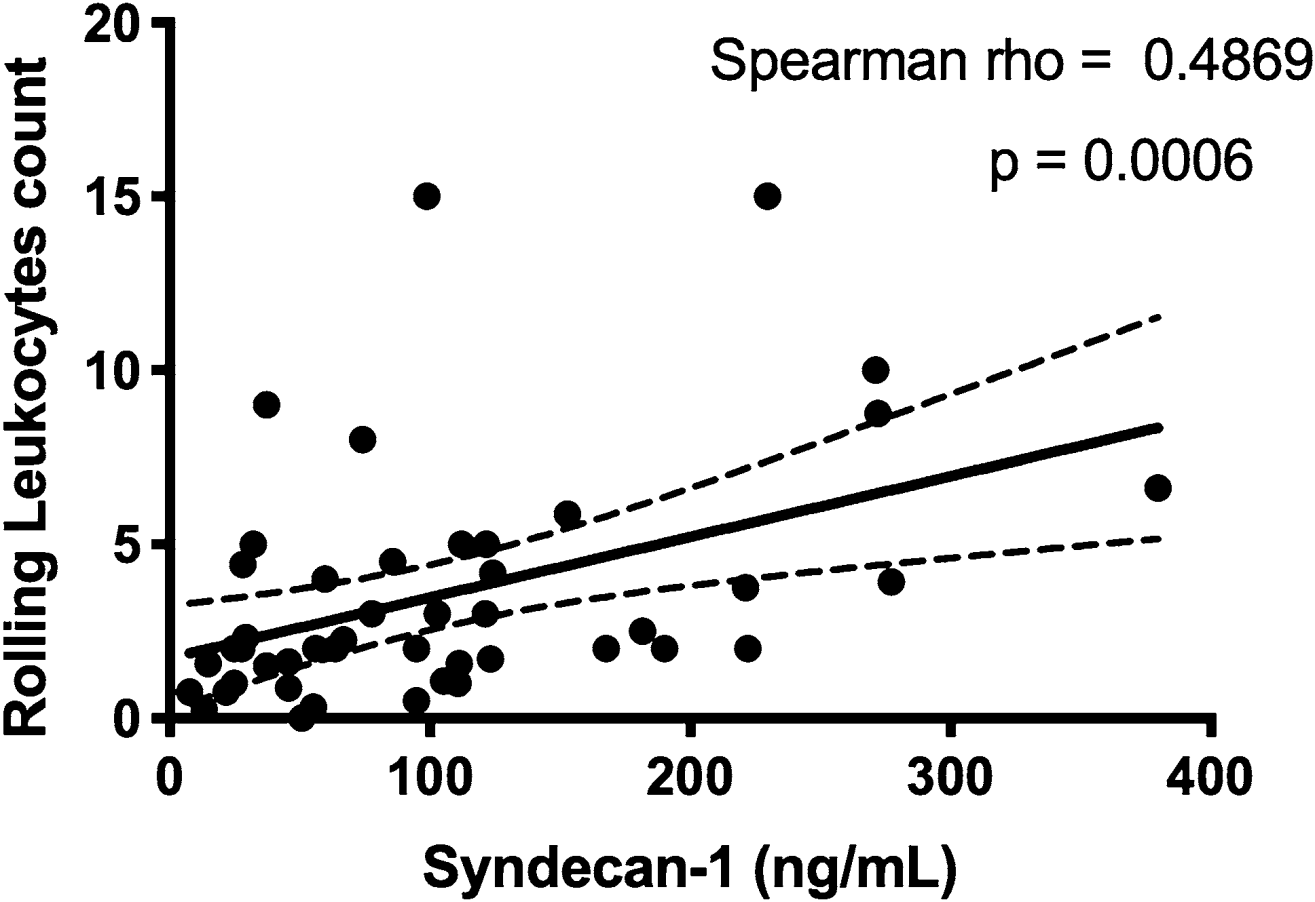
Correlation between Syndecan-1 and sublingual microcirculatory rolling leukocytes. Syndecan-1 levels and the sublingual microcirculatory rolling leukocyte counts show a significant but moderate correlation.

Glycocalyx degradation was demonstrated by significantly increased levels of syndecan-1. Syndecan-1 increased from T0 to T1 (42 [25–56] vs*.* 107 [86–164] ng/mL, p < 0.001) and remained increased at T2 when compared to baseline (42 [25–56] vs*.* 158 [102–240] ng/mL, *p* < 0.001) (Fig. 8). In Fig. 9, a significant but moderate correlation between the sublingual microcirculatory rolling leukocytes and the syndecan-1 levels was demonstrated (R = 0.4869, *p* = 0.0006).

## Discussion

The present study showed that the observed microcirculatory alterations were accompanied by reduction of convection parameters (MFI and PPV), activation and recruitment of leukocytes during the perioperative period regardless of macrocirculatory variables. Secondly, the increase in leukocyte–endothelial interactions was associated with alterations of sublingual microvascular blood perfusion 24 h postoperatively. Thirdly, the increase in rolling leukocytes in the sublingual area was corroborated by glycocalyx degradation.

Hemodynamically, all the patients were stable during the study period. However, at the end of surgery and at 24 h after surgery, we found elevated lactate levels likely due to a combination of tissue hypoxia resulting from decreased tissue perfusion, and disability of the remnant liver to metabolize the lactate. Moreover, low arterial haemoglobin oxygen saturation was also observed within 24 h after surgery. These results indicate that the microcirculatory dysfunction and impairment of oxygenation can predominantly occur even until 24 h after major liver surgery as was shown by the convection parameters (MFI and PPV) that had also deteriorated in sublingual area.

Monitoring of the microcirculation at the sublingual area is preferred due to its easy accessibility. However, controversy exists whether the sublingual microcirculation is representative of the microcirculation in other organ surfaces^26,27^. In the current study, no microvascular flow or density alterations were found intraoperatively in the sublingual area, nor on the intestinal surfaces showing that both organ sites were reciprocal. However, as mentioned above, we showed that sublingual microcirculatory perfusion at 24 h after surgery indicated by PPV and MFI, were predominantly altered likely due to redirection of blood flow to protect internal organs after the ischemic, inflammatory and surgical impact.

In the current study, alterations of sublingual MFI were observed post-operatively, coinciding with a peak in rolling microcirculatory leukocyte counts. Activated leukocytes adhere to the venule wall, which during severe inflammation may form clumps and eventually grow to occlude blood flow^28^. Furthermore, leukocyte extravasation leads to increased vascular permeability causing edema and thereby, reduction of the oxygen diffusing capacity^29^. Hypoxia, as shown in the present study by high lactate levels 24 h after surgery, resulted from the reduced capability of the microcirculation to supply oxygen. Oxygen depletion has shown to induce the expression of hypoxia inducible factor- 1a (HIF-_1a_)^30^, which in turn perpetuates leukocyte activation, thereby creating a positive feedback loop in which hypoxia and inflammation mutually complement each other. Interestingly, the sublingual MFI was at 24 h after surgery lower than the point-of-care threshold of MFI of 2.6^15^. This threshold for MFI was established in clinical studies and a recent consensus guideline for microcirculatory assessment, after it was demonstrated that a sublingual MFI lower than 2.6 correlated with high morbidity and mortality^15,31,32^. In the present study involving patients undergoing high-risk surgery, 63% of the patients had a severe complication (Clavien–Dindo ≥ 3) within 30 days after surgery, and indeed the MFI fell below this threshold at 24 h after surgery. The hepatic microcirculation however, showed a higher MFI at the end of surgery. However, this different finding in the liver is caused by the greater volume of inflowing blood in the remnant liver after resection at the end of surgery. Unfortunately, the sample size was too small to show any correlation between the MFI and the individual outcome data of the patients.

The ability to study leukocyte activation allows the direct evaluation of the inflammatory status of the patient following surgery. In the current study, the peak number of non-rolling sublingual microcirculatory leukocytes at the end of surgery, was followed by a peak of rolling sublingual microcirculatory leukocytes 24 h post-operatively. This is in accordance with the study of Efron et al., in which in 15 major hepatectomy patients, the highest concentration of activated monocytes was observed at post-operative day (POD) 1^33^. Additionally, the authors were able to detect a significant increase in the number of non-activated monocytes following resection (before abdominal closure) followed by a decrease at POD1 demonstrating the inverse behaviour compared to their activated counter parts. Furthermore, a study investigating the clinical impact of perioperative monitoring of the immune response during hepatectomy revealed that a postoperative peak in systemic leukocyte numbers was significantly associated with adverse events following surgery^34^. Therefore, changes in leukocyte numbers are a potential surrogate marker for identifying patients who may benefit from prophylactic therapy or closer monitoring during the ICU period^6,35^. Interestingly, the patients exposed to longer VIO duration showed significantly higher levels of rolling leukocytes and lactate values. This important finding shows that IRI is a strong trigger for rolling leukocytes and opens the door for future research to gain more detailed information on IRI-induced phenomena. The present study has shown the ability to assess rolling leukocytes, real-time at the bedside of the patient in the sublingual area, which is a potential additional parameter for the clinical use in critically ill patients.

The identification of leukocyte adhesion and activation potentially contributes to the diagnosis of vascular pathology and provides important clinical feedback for optimising treatment strategies. The present study showed a moderate correlation between sublingual microcirculatory rolling leukocytes and glycocalyx degradation (Syndecan-1). In an experimental study by Constantinescu et al. it was shown that the endothelial glycocalyx modulates the immobilization of leukocytes^12^. The glycocalyx has an anti-adhesive character, and protects the endothelium from interaction of the cells carried in the bloodstream. A hallmark of leukocyte activation is cell adhesion to the endothelium for subsequent diapedesis^14,36,37^. Degradation of the endothelial barrier must occur in order for expression of adhesion molecules on the endothelial layer. Visualisation of leukocyte activation therefore, is a direct manifestation of endothelial barrier dysfunction as a result of glycocalyx degradation^12^. Although the present study only examined Syndecan-1 levels, there are other factors such as selectins, PECAM and chemokines that can be involved in the process of leukocyte activation and transmigration. There is need for more research to identify inflammatory factors to allow for a more precise correlation between the rolling leukocytes and glycocalyx degradation markers.

Our study has several limitations. Firstly, as aforementioned, the small sample size is a limitation of the present study, as correlations between the primary outcome parameters and individual patient outcomes were lacking, although the overall outcome corresponded with glycocalyx degradation and decreased MFI. The same limitation also applies to the two groups of short and long cumulative VIO duration; whereas the long-VIO group showed significantly higher levels of rolling leukocytes, the Syndecan-1 levels were not different. More research is needed with larger sample sizes to gain additional information on physiological microcirculatory cell (red and white blood cells) kinetics and endothelial cell injury. Also, data regarding intraoperative application of glucocorticoids, type of fluid replacement and usage of blood products are important factors influencing inflammation and also leukocyte activation. This data is not discussed in the present study, and should be investigated in future research. Another limitation is the inability of the current HVM and software methodology to assess the hepatic microcirculatory leukocytes. Intensive device and software development are required to overcome this shortcoming.

In conclusion, we demonstrated that the impairment of perfusion and oxygenation occurs in 24 h after hepatectomy despite the improvement of hemodynamic variables. The microcirculatory perfusion was characterized by low convection capacity and high number of rolling leukocytes. The ability to sublingually monitor the rolling behaviour of the microcirculatory leukocytes allows for early identification of patients at risk of increased inflammatory response following hepatectomy.

## Methods

A single center, prospective observational study evaluating the impact of major liver resection on local and distant organ microcirculation. The study was performed peri-operatively at the Department of Surgery, Amsterdam UMC, location Academic Medical Center, University of Amsterdam, The Netherlands. Written informed consent was obtained from all patients prior to surgery, and all procedures carried out within this study were in accordance with the Declaration of Helsinki. The study was given ethical consent by the ethical committee at the Amsterdam UMC (ethical committee reference number 2016-256).

### Study design

A baseline hepatic microcirculatory measurement was made at the beginning of surgery after laparotomy (T0); on the liver (the intended liver remnant), intestines and sublingual area. A second microcirculatory measurement was made at the end of surgery just before skin closure (T1), again on the same organ surfaces. 24 h postoperatively (T2) only the sublingual microcirculation was assessed to monitor microcirculatory alterations.

### Hepatic vascular inflow occlusion (VIO)

VIO may be undertaken in major liver resections to reduce blood loss. VIO was performed using a tourniquet around the hepatoduodenal ligament with which blood flow through the hepatic artery and the portal vein towards the liver can be temporarily arrested. Intermittent VIO was applied during parenchymal liver transection by vascular occlusion for at least 20 min (ischemia phase), followed by 10 min of reperfusion (reperfusion phase). The cumulative VIO (ischemia) duration was calculated for all patients in minutes. Three groups of VIO duration were defined; no VIO (0 min), short-VIO (1–30 min), long-VIO (31–70 min).

### Surgical and postoperative information

The included patients undergoing liver resection were investigated during surgery and 24 h later.

Surgical information (duration of surgery, blood loss, VIO) and postoperative events (morbidity and mortality) were recorded of all of the participating patients. For major complications occurring within 30 days postoperatively, the Clavien-Dindo score ≥ 3 was used^38^. Morbidity was defined as any occurrence of any complication within 24 h or 30 days. 30-day mortality was defined as occurrence of death within 30 days postoperatively.

### Clinical laboratory examinations

Blood gas assessments were performed as a part of hospital routine protocol. Parameters of haemoglobin concentration and percentage of oxyhaemoglobin were assessed as important indicators of perfusion. Lactate concentrations and liver transaminases were also assessed peri-operatively as indicators of liver damage. Number of systemic leukocytes was assessed to compare with the microcirculatory leukocyte values.

### Non-invasive assessment of organ microcirculation

Organ microcirculation assessments were performed using Cytocam-IDFI (Braedius Medical, Huizen, The Netherlands), the third and latest generation of HMVs^39^. Image sequences generated by Cytocam-IDFI were captured using CCTools 1.7.12 (Braedius Medical, Huizen, The Netherlands) and stored on a powerful medical grade computer. Image sequences were captured at 25 frames/s; 3 consecutive image sequences of 4 s were captured per time-point per organ surface to minimise heterogeneity in the microscope field of view. Tip of the Cytocam probe contains light emitting diodes that expel green light at 530 nm. This is within the absorption range of haemoglobin allowing it to appear dark on the screen. A resolution of 2208 × 1648 pixels covers 1.242mm^2^ of tissue in the field of view. These images are magnified with factor 4, and allow more capillary visualisation^39^.

For assessment of sublingual microcirculation, a disposable microscope cap (Braedius Medical, Huizen, The Netherlands) was placed on the tip of the Cytocam probe and placed in the sublingual cave adjacent to the lingual frenulum on either the right or left side. Video captures were made when the projected image sequences were deemed stable, free from artefacts (such as saliva and pressure) and individual red blood cells (RBCs) were visible in the capillaries.

During intestinal and hepatic microcirculatory measurements, the Cytocam was covered with a sterile disposable microscope cap (Braedius Medical, Huizen, The Netherlands) and covered with a laparoscopic camera cover (Camera Cover, Microtek Medical B.V Zutphen, the Netherlands) before entering the sterile operative field. During image acquisition excess blood was wiped off with a sterile gauze. The Cytocam probe was placed perpendicular to the organ surface, pressure was relieved before image sequences were captured. The microcirculatory assessments were performed on the serosal surface of the jejunum 50 cm distally from the ligament of Treitz with at least 1 large vessel in the field of view. The hepatic measurements were assessed on segment 3 or segment 6, according to the future liver remnant after planned right or left sided hemi-hepatectomy. Hepatic and intestinal microcirculatory alterations were monitored before and after hepatic resection; the first measurement (T0) at the beginning of surgery after laparotomy, and the second measurement (T1) at the end of surgery just before skin closure.

### Analysis of organ microcirculation

All microcirculatory analyses were blindly performed by the Automated Vascular Analysis (AVA) 3.2 software (MicroVision Medical, Amsterdam, The Netherlands). Only vessels < 20 µm were analysed according to the most recent consensus guideline^15^. Two main functional parameters in the microcirculation governing convection and diffusion were analysed^17^; (1) the convection parameters; Mean Flow Index (MFI) [arbitrary units] and Portion of Perfused Vessels (PPV) [%] quantitatively describes the behaviour of red blood cell flow seen within the microcirculation, (2) the diffusion parameters; Total Vessel Index (TVD) [mm/mm^2^] and Perfused Vessel Density (PVD) [mm/mm^2^]. The MFI was determined using a grid-based system of which the analysis screen is divided into quadrants, in which a value governing the predominant flow was given (0 = no flow, 1 = intermittent flow, 2 = sluggish flow, 3 = continuous flow). The final MFI score was automatically calculated as the average score of all quadrants. The PPV was automatically determined by the software as a binomial expression of overall red blood cell velocity following flow classification of individual vessels (according to MFI flow classification). The TVD was calculated as the sum of the length of all capillaries in the field of view. The PVD was determined as the density of the micro-vessels in the field of view (i.e. TVD) multiplied by the PPV. See Table 5 for a summary of the Cytocam operating steps and the data analysis using AVA software.

**Table 5:** Workflow: from Cytocam assessments to analysis using AVA software.

Steps	Cytocam device operating procedures and analysis using AVA software
Device set-up	The Cytocam imaging is set-up and attached to the computer.
CC-tools software started	The CC-tools software embedded in the computer attached to the Cytocam is started and ready to capture microcirculatory video images.
Device ready to record	The Cytocam device directly records the video images on the hard disc of the computer that it is coupled to. The green START button is active and ready to record.
Disposable cap	The imaging probe of the Cytocam device is covered with a sterile plastic disposable cap and this is confirmed in the CC-tools software before start of the capture
Probe placed on the ROI	The imaging probe of the Cytocam device must be placed on the region of interest (ROI). The tip of the imaging probe must make sure to gently touch the preferred tissue surface, avoiding pressure on the microvascular system.
Focus	During the assessment, the focus is adjusted by the CC-tools software. After assessing a sharp image, the imaging probe of the Cytocam device must be stabilized for recording a minimum of 4 s of stable video footage per adjacent location.
Video-images are stored	The assessed video images by Cytocam are captured by the CC-tools software and is set to analyse, store and catalogue the obtained data on hard disk. The data can be exported now to analyse data also in other dedicated software programs.
Automated vascular analysis (AVA) software	The video-clips are exported as AVI files in CC-tools software, and opened in the AVA software to analyse the microcirculatory flow, density and leukocyte parameters. The steps of the semi-automatic analysis are explained in the handout of the software.
Data output parameters	The following output parameters are assessed by the AVA software: 1) **TVD**, Total Vessel Density: the ratio of the total length of the microvascular vessels and the total area. This parameter shows the morphological density. Unit: mm/mm^2^ 2) **PVD**, Perfused Vessel Density: the TVD with the correction of the actual perfused vessels, the software calculates only the vessels with sluggish or continuous flow, and discards the vessel with no flow, stop flow and intermittent flow. The PVD shows the functional density, also the measure for the Functional Capillary Density (FCD). Unit: mm/mm^2^ 3) **PPV**, Percentage of Perfused Vessel: Percentage of the vessel with a continuous or sluggish flow. Unit: % 4) **MFI**, Microcirculatory Flow Index: the average flow score, the software divides the total area in 4 quadrants, each quadrant is scored by the observer for; 0 no flow, 1 intermittent flow, 2 sluggish flow, 3 continuous flow. The algorithm embedded in the software calculates an average score between 0 and 3. 5) **Rolling, non-rolling and total leukocytes**: average of the count of white bands generated in the space–time diagram application (Fig. 10), in at least 4 capillary post-capillary venule units. Unit: (Ls)/C-PCV/4secs

### Microcirculatory leukocyte identification and quantification

**Figure 10:**
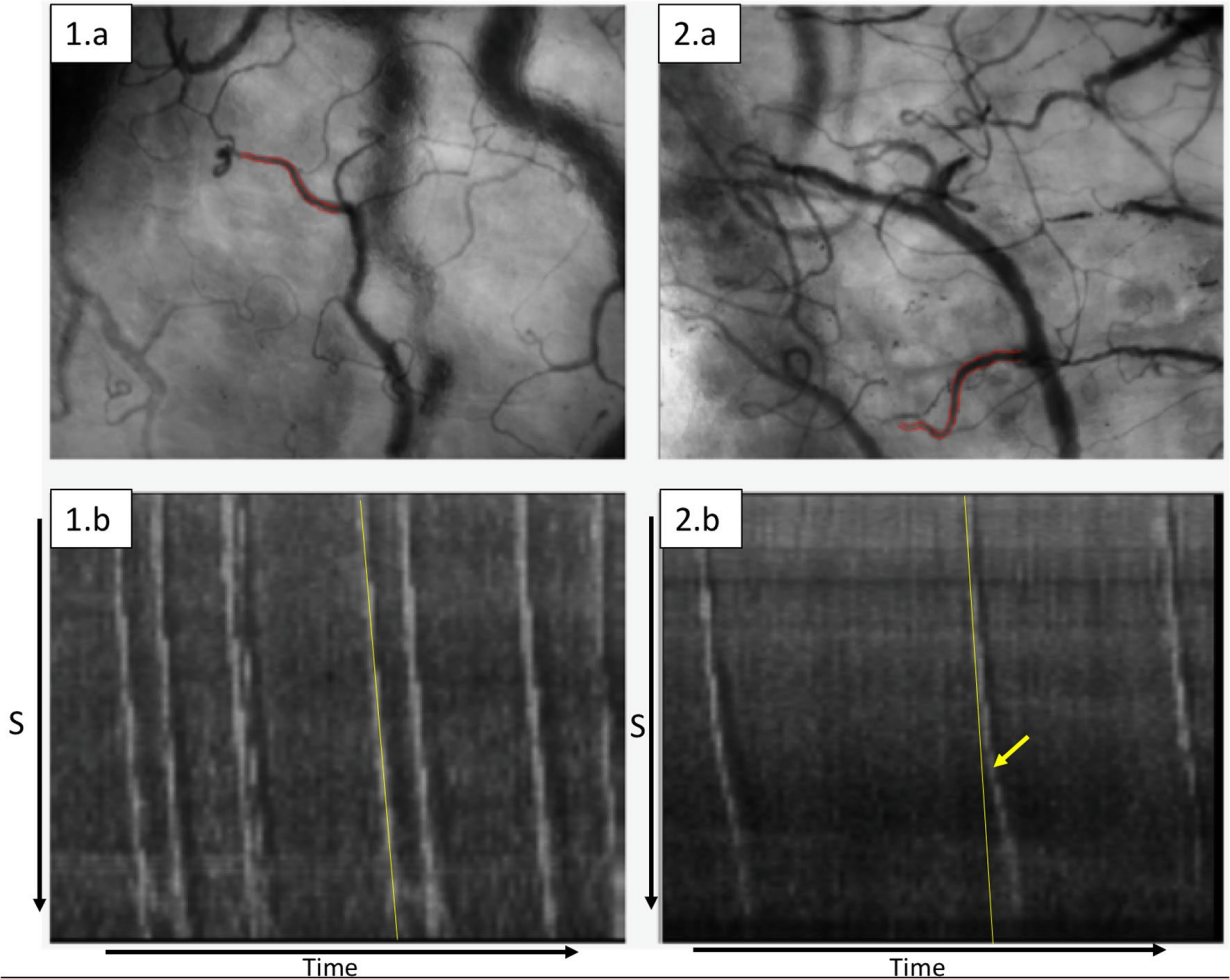
Screenshots of sublingual microcirculation obtained by incident dark-field imaging and examples of space–time diagrams generated from the microvascular units indicated in red in the images. (**1.a**) Example of sublingual microcirculation. In this image, one capillary-post-capillary venule unit is shown by the redlined outlines of the vessel walls. Dimensions of the field of view of the sublingual microcirculation screenshot is 1.55 mm × 1.16 mm. (**1.b**) Shows the generated space–time diagram of the capillary-post capillary venule. The space–time diagram shows several straight white bands, indicating non-rolling leukocytes flowing through the capillary and post capillary venule without rolling or contact with the vessel wall. The white band is a straight line, corresponding with the straight yellow, control line. (**2.a**) Example of sublingual microcirculation. In this image, one capillary-post capillary venule unit is shown by the redlined outlines of the vessel walls. Dimensions of the field of view of this sublingual microcirculation screenshot is 1.55 mm × 1.16 mm. (**2.b**) Shows the generated space–time diagram of the capillary-post capillary venule. The space–time diagram shows several white bands that are not straight, indicating rolling leukocytes flowing through the capillary and getting into contact with the endothelial wall in the post capillary venule part. The white band is slanted, and the straight yellow line drawn on it, does not fit the white band of this rolling leukocyte. The yellow arrow shows the start of the slanting of the white band, this is also the moment at which the white band is not straight anymore due to significant change in the velocity of the leukocyte by rolling or adherence to the endothelium. *S* the axis of the space–time diagram for space, *Time* the axis of the space–time diagram indicating the time.

A detailed description of microcirculatory leukocyte quantification was described in our previous study^21^. Leukocytes were identified as either rolling or non-rolling using Space–time diagrams (STDs). The STD is one of the applications within the AVA 3.2 software (MicroVision Medical, Amsterdam, The Netherlands), which was originally developed for the assessment of red blood cell velocity. The STD is a typical diagram with an y-axis and an x-axis; the y-axis corresponds to the length of the vessel and the x-axis to the time lapse. The STD shows the time (duration of the video-clip in terms of frame numbers, x-axis) that a RBC needs to flow over a space (length of the vessel, y-axis). The STD is a diagram consisting black and white lines that are alternating; the black bands represent the red blood cells, the white bands however are plasma gaps or leukocytes. The delta (slope) of each band represents the velocity of that band. Leukocytes were identified as a bright white band in the STDs immediately followed by a dark wide black band, which is caused by accumulation of RBCs behind the slower flowing leukocytes inside the capillary, see Fig. 10. The accumulation of the red blood cells behind the leukocytes offers important visual confirmation and allows to distinguish between plasma gaps or leukocytes. A leukocyte is a large, heavy cell, whereas a plasma gap represents fluid changing of shape being a rheological medium for blood to flow. Thereby, a plasma gap is always a smaller white band and is not followed by a larger black band. Rolling and non-rolling leukocytes were differentiated visually, by identification of a large white band which is directly followed by a large black band (red blood cell accumulation).

The STD was assessed using the anatomical units of the microcirculation that were previously validated to identify and quantify microcirculatory leukocytes^21^. These anatomical units consist of a capillary which merges into a small venule distal to the capillary (post-capillary venule) without branching. The capillary, post-capillary venule (C-PCV) unit allows visualization of the leukocyte first in the capillary, and later in the post-capillary venule in which the rolling of the leukocytes occur. See Fig. 10 for an example of a C-PCV unit in the sublingual microcirculation, and the STD generated from this vessel segment.

### The determination of rolling and non-rolling leukocytes

A leukocyte was determined to be rolling when there was a distinct deviation in the slope of the white band entering from the capillary to the post-capillary venule. This marks the slowdown of the leukocytes caused by the interaction of the selectins and integrins during inflammation. The white bands that represent a rolling leukocyte are thereby never straight, and show after entering the PCV, a significant slowdown with a visual slanting of the band (see Fig. 10-2b) *.

Non-rolling leukocytes were identified as bright white bands, linear in appearance when transitioning from the capillary to the post-capillary venule (see Fig. 9). These bands are always straight, and always have an angle of 180°. By drawing a straight line over the white band, it is possible to control the straightness of the band. If this straight line is not fitting the white band, the band is having an angle more or less than 180° (see Fig. 10-1b).

The total leukocyte quantification was calculated as the sum of non-rolling and rolling leukocytes. 4 capillary post-capillary venule units were counted per 100 frame image sequences, which corresponds to 4 s. An average was taken per clip and expressed as leukocytes (Ls)/C-PCV/4 s.

The microcirculatory leukocytes were assessed in the intestinal and sublingual organ surfaces. As the STD methodology was only validated in C-PCV units, it was not possible to assess the microcirculatory leukocytes in the sinusoidal hepatic microcirculation.

### Glycocalyx degradation

Blood samples were collected at T0, T1 and T2 and stored at − 80 °C. Syndecan-1 levels were measured by enzyme-linked immunosorbent assay (ELISA) as a parameter of glycocalyx degradation^40^. Blood plasma was analysed at a 50-fold dilution. Syndecan-1 levels in the blood plasma were measured with a commercially available ELISA kit for Human syndecan-1 (R&D system, DY2780, Minneapolis USA).

### Statistical analysis

Values are expressed as mean ± SD when normally distributed (Shapiro–Wilk test), or as median [IQR] otherwise. The normal distributed dependent continuous data were compared using a Students t test for two time points or One-way analysis of variance (ANOVA) for more than two time-points, the non-normally distributed continuous data were compared using a Wilcoxon or Friedman test. Categorical data were analyzed using the Fishers-exact test. Student t test or ANOVA was performed for independent variables in normal distributed data, otherwise Mann–Whitney or Kruskal–Wallis test were used. The mixed model analysis was used if there are missing values. Correlation analysis was assessed using the Spearman rho correlation coefficient. A p value of < 0.05 was considered statistically significant in the present study. Statistical analysis was performed using IBM SPSS software version 24.0 [SPSS, Chicago (IL), United States] and GraphPad PRISM 8.0 (GraphPad Software, La Jolla, CA, USA).
